# Benefits and barriers among volunteer teaching faculty: comparison between those who precept and those who do not in the core pediatrics clerkship

**DOI:** 10.3402/meo.v18i0.20733

**Published:** 2013-05-03

**Authors:** Michael S. Ryan, Allison A. Vanderbilt, Thasia W. Lewis, Molly A. Madden

**Affiliations:** 1 Department of Pediatrics, Children’s Hospital of Richmond, Virginia Commonwealth University, Richmond, VA, USA; 2 School of Medicine, Center on Health Disparities, Virginia Commonwealth University, Richmond, VA, USA

**Keywords:** preceptor, pediatric, community, pediatric clerkship, undergraduate medical education

## Abstract

**Background:**

Community-based outpatient experiences are a core component of the clinical years in medical school. Central to the success of this experience is the recruitment and retention of volunteer faculty from the community. Prior studies have identified reasons why some preceptors volunteer their time however, there is a paucity of data comparing those who volunteer from those who do not.

**Methods:**

A survey was developed following a review of previous studies addressing perceptions of community-based preceptors. A non-parametric, Mann–Whitney *U* test was used to compare active preceptors (APs) and inactive preceptors (IPs) and all data were analyzed in SPSS 20.0.

**Results:**

There was a 28% response rate. Preceptors showed similar demographic characteristics, valued intrinsic over extrinsic benefits, and appreciated Continuing Medical Education (CME)/Maintenance of Certification (MOC) opportunities as the highest extrinsic reward. APs were more likely to also precept at the M1/M2 level and value recognition and faculty development opportunities (*p*<0.05). IPs denoted time as the most significant barrier and, in comparison to APs, rated financial compensation as more important (*p*<0.05).

**Conclusions:**

Community preceptors are motivated by intrinsic benefits of teaching. Efforts to recruit should initially focus on promoting awareness of teaching opportunities and offering CME/MOC opportunities. Increasing the pool of preceptors may require financial compensation.

## Introduction

In recent years, there has been a shift in emphasis of undergraduate medical education from the inpatient to outpatient/primary care-based settings (1–5). As a result, almost all medical schools in the United States and Canada provide an office-based primary care experience as a part of their core clerkships (4, 6). Medical students benefit greatly from this experience as they are provided the opportunity to expand not only their knowledge of patient care, but also formulate an understanding of the healthcare delivery system including the medical home model and the business of medicine (2, 5). Accordingly, community-based, outpatient experiences have been identified as a requirement of several accreditation and professional organizations, such as the Association of American Medical Colleges (AAMC) (7), Liaison Committee on Medical Education (LCME) (8), and the Council on Medical Student Education in Pediatrics (COMSEP) (9).

Although there is pressure from the AAMC, LCME, and COMSEP to provide community-based rotations for medical students, there are several challenges to overcome. Among the greatest challenges are recruitment and retention of faculty preceptors within the community (9, 10). Specific barriers include changes in the healthcare system and impediments to clinical productivity resulting in the potential for reduced reimbursement (11–14). With the high demands on community-based physicians, preceptors struggle with finding time for teaching because of the constraints related to managed care and lack of flexibility in scheduling (9, 10, 14). Because of these barriers, previous studies have attempted to identify motivations of community faculty in an attempt to assist in future recruitment and retention.

Previous literature has identified intrinsic and extrinsic motivators that encourage physicians to serve as preceptors. Intrinsic motivators include satisfaction from sharing knowledge, demonstrating the primary care model, interacting with other volunteer faculty, and directly participating in the education of future physicians (4, 11, 15–21). Extrinsic motivators include direct financial compensation, awards, recognition, and access to university resources (22, 23). Several studies have demonstrated the superiority of intrinsic over extrinsic motivators in the recruitment and retention of community preceptors (4, 24, 25).

While prior studies have identified reasons why active preceptors (APs) choose to volunteer their time (11, 15, 24), there is only one study which explicitly surveyed inactive preceptors (IPs) to identify barriers and compare this group to their active peers (26). That study surveyed family medicine physicians in Canada and identified practice-related constraints and unawareness of teaching opportunities as important barriers and found that graduates of the local medical school and/or residency program were more likely to volunteer their time. To date, no previous studies have sought to identify differences in preferred incentives among currently active and currently IPs.

The purpose of this study was to survey all community-based pediatric physicians surrounding an urban academic center in the United States to determine why some pediatric physicians volunteer to work with medical students and others do not. Our research question was; what differences exist between APs and IPs of third-year medical students rotating through pediatrics? We sought to specifically identify differences between the two groups in terms of (1) demographic variables (2), teaching responsibilities, and (3) incentives/rewards. We also sought to identify perceived barriers among IPs. For the purpose of this study, we defined APs as those who had precepted at least one M3 student over the preceding 12 months.

## Methods

This survey design study was administered in January 2012. The participants in this study were identified using an internal departmental database of known pediatricians in the Richmond, Virginia metropolitan area. Surveys were distributed by mail as well as email to all known listings on the database (*N*=340). This study received approval from the Internal Review Board at Virginia Commonwealth University.

### Survey instrument

The survey was developed following review of previous studies addressing perceptions of community-based preceptors (11, 21, 22, 24). We added additional demographic questions specific to our region including practice location (i.e., downtown, near suburbs, etc.) and ownership (i.e., private practice vs. specified hospital system). The final survey consisted of 50 questions addressing the following domains: participant’s demographics, current involvement in teaching, perceived motivations/rewards, and barriers to precepting.

Demographic questions included characteristics of the individual physician’s age, gender, specialty, practice environment (ownership, part-time/full-time status and location), as well as his/her training (medical school and residency locations and specialty). Current teaching questions asked how many learners the physician had worked with in each of the following settings over the past 12 months: pre-clinical years, M3 year, and post-graduate/residency years. Questions about motivations and incentives required physicians to rate existing or potential intrinsic (i.e., ‘opportunity to share knowledge with students’) and extrinsic (i.e., ‘financial compensation’) rewards on a scale of 1–4 (1 = not important; 4 = very important). Finally, the barriers section asked IPs to rate the significance of various barriers to precepting (i.e., ‘lack of time’) on a scale of 1–4 (1 = not at all significant; 4 = very significant).

### Data collection

Study data were collected and managed using REDCap electronic data capture tools hosted at Virginia Commonwealth University (27). REDCap (Research Electronic Data Capture) is a secure web-based application designed to support data capture for research studies, providing:

1. an intuitive interface for validated data entry;
2. audit trails for tracking data manipulation and export procedures;
3. automated export procedures for seamless data downloads to common statistical packages; and
4. procedures for importing data from external sources. Data obtained from receipt of mail surveys were entered into the REDCap database by the primary investigator.

Descriptive statistics were calculated for the participants that who worked with an M3 student(s) in the past 12 months (AP) and those who had not worked with a student in the past 12 months or more (IP).

To assess the internal consistency for the 50-item Pediatric Preceptor Survey, Cronbach’s alpha was calculated (*α*=0.927). A non-parametric, Mann–Whitney *U* test was used to compare the IP and AP groups’ mean scores and standard deviations were calculated, and frequency data related to demographic information. All data were analyzed in SPSS 20.0.

## Results

There were 340 surveys distributed (182 by email and 158 by postal mail). In total, 34 emails were marked as ‘delivery failures’, 25 letters were returned due to address changes, and 20 surveys were incomplete resulting in 261 successful deliveries. Overall, 72 of the 261 (28% response rate) surveys were completed, and therefore used in the analysis. Demographic data such as age, gender, and ethnicity for APs and IPs are shown in Table 1.

**Table 1:** Participant demographics

Characteristic (%)	Overall (*N*=72)	Inactive (*n*=36)	Active (*n*=36)
Female	56.3	60.0	52.8
Age			
30–40	15.3	19.5	11.1
41–50	36.1	27.8	44.5
51–60	27.8	22.2	33.4
> 60	20.8	30.6	11.1
How would you best identify yourself?			
General outpatient pediatrician	91.7	91.4	94.4
General outpatient pediatrician with additional training/specialty (i.e., development, sports medicine)	6.9	8.6	5.6
How would you characterize your practice?			
Solo practice	8.5	8.6	8.3
Small group practice < 5 pediatricians in practice	46.5	54.3	38.9
Large group practice > 5 pediatricians in practice	45.1	37.1	52.8
How would you characterize the ownership of your practice?			
Private practice	98.6	100.0	97.2
Other	1.4	0.0	2.8
How many days a week do you work?			
< 2	0.0	0.0	0.0
2–3	16.7	22.2	11.1
> 3	83.3	77.8	88.9
Where is your practice?			
Downtown Richmond	2.8	0.0	5.6
City of Richmond, outside Downtown	12.7	20.0	5.6
Near suburbs of Richmond (i.e., West End, Henrico, Midlothian, East End)	59.2	57.1	61.1
Other suburb/county in greater Richmond/Petersburg area	22.5	17.1	27.8
Other city/county (Fredericksburg, NOVA, Williamsburg, etc.)	2.8	5.7	0.0
Where did you attend medical school?			
VCU/MCV	35.2	34.3	36.1
Other Virginia medical school	14.1	17.1	11.1
Medical school outside Virginia	50.7	48.6	52.8
Where did you complete residency training?			
VCU/MCV	61.1	52.8	69.4
Other Virginia residency training program	1.4	2.8	0.0
Residency training program outside Virginia	37.5	44.4	30.6
What type of residency training did you complete?			
Pediatrics	93.1	88.9	97.2
Medicine/pediatrics	4.2	8.3	0.0
Family medicine	2.8	2.8	2.8
Other	0.0	0.0	0.0

*Note*: Percentages may not total because of rounding.

Percentages of preceptors working with students in the pre-clinical years and pediatrics residents are shown in Table 2. APs were more likely to also precept an M1 and/or M2 student as compared to IPs. There was no significant difference between APs and IPs in terms of their likelihood to also precept pediatrics residents.

**Table 2:** Comparison of active and inactive preceptors’ motivations to precept students

	Inactive (*n*=36)		Active (*n*=36)		Mann–Whitney
			
Survey item	*M*	*SD*	*M*	*SD*	*U*	
Intrinsic factors					
Opportunity to demonstrate the primary care model to students	3.53	0.629	3.58	0.500	534.0
Opportunity to share knowledge with students	3.60	0.498	3.64	0.487	519.0
Opportunity to participate in the education of the next generation of doctors	3.67	0.547	3.61	0.494	499.0
Opportunity to interact with other primary care educators	2.03	1.033	2.28	1.162	481.0
Extrinsic factors					
Faculty status at VCU	2.07	0.944	2.14	1.018	521.0
Access to resources at VCU (library, grand rounds, etc.)	1.77	1.073	1.922	1.105	504.0
Financial compensation	2.30	0.877	1.86	1.192	363.5*
CME credit	2.64	1.194	2.89	1.008	525.5
Able to apply participation to MOC requirements	3.00	1.078	3.11	1.036	544.0
VCUHS parking sticker	1.94	1.116	1.97	1.207	589.5
Opportunity for an ‘expedited’ admissions process for your patients	1.74	1.064	2.19	1.091	415.5
Discounted tuition at VCU	1.91	1.146	2.03	1.218	510.0
Discounted access to VCUHS health/fitness center(s)	1.39	0.715	1.80	1.132	447.5
Discounted tickets to VCU campus events (concerts, athletic events, etc.)	1.78	0.906	2.09	1.067	473.5
A VCU email account	1.06	0.246	1.53	0.929	412.0*
Annual recognition lunch/dinner	1.31	0.592	1.71	0.938	425.0
Letter of appreciation	1.62	0.793	2.26	1.010	359.5*
Plaque/certificate of appreciation	1.53	0.671	2.09	1.040	396.0*
Feedback regarding your performance (med student evaluations)	2.61	0.933	2.91	1.138	453.0
Periodic funded social gatherings among community preceptors	1.50	0.803	2.21	1.008	328.0*
Training/workshops on education (how to teach effectively, give feedback, evaluate)	1.82	0.950	2.40	1.117	406.5*
Opportunity to receive a teaching award	1.53	0.776	2.14	1.061	355.0*

*Note*: The scale ranged from ‘1’ being not important to ‘4’ being very important.

*M*=mean; SD = standard deviation; and *U*=Mann–Whitney *U*.

* *p*<0.05.

Mean scores and standard deviations for both groups and by item for the pediatric preceptor survey are shown in Table 3. As shown, comparison between groups revealed that participants in the AP group reported significantly higher ratings of the following incentives: letter of appreciation, plaque/certificate, social gatherings, teaching workshops, and opportunity to receive a teaching award; whereas the IP group reported significantly higher ratings only to financial compensation.

**Table 3:** Additional teaching efforts of active vs. inactive preceptors

	Inactive	Active	Mann–Whitney *U* test *p*
Precepts M1 and/or M2 student(s) (%)	30.6	55.6	0.033*
Precepts pediatrics resident(s) (%)	13.9	16.7	0.745

* *p*<0.05.

**Fig. 1:**
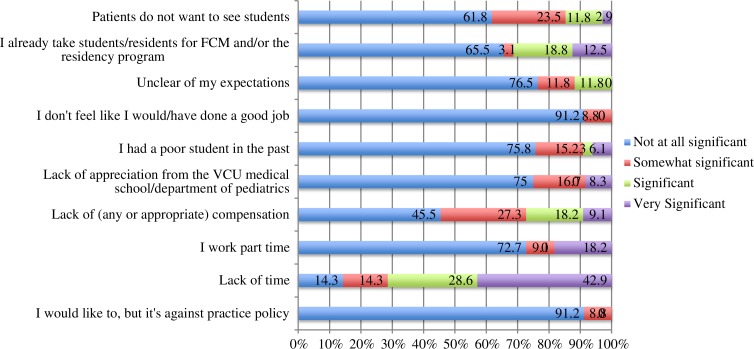
Non-preceptor barriers in decision to precept students. *Note*: *N*=36.

IPs identified barriers to why they do not precept students. Fig. 1 demonstrates the range of not significant to very significant barriers to precept M3 students. The least significant barrier was that it was against practice policy to precept medical students; whereas the most significant barrier was lack of time.

## Discussion

This study was conducted to answer our research question: what differences exist between APs and IPs of third-year medical students rotating through pediatrics? We found no demographic variables that differed significantly between the two groups. APs were, however, more likely to also precept students in the pre-clinical years. In comparing incentives, both groups valued intrinsic benefits; however, APs showed a significant preference toward developmental opportunities and appreciation while IPs showed a significant preference toward financial compensation. Finally, IPs reported ‘lack of time’ as the most significant barrier to precepting students.

## Rewards

Both APs and IPs endorsed intrinsic benefits as the highest overall motivation for accepting potential students. This is consistent with a large body of literature demonstrating the altruistic motivation among community preceptors (4, 11, 15–22, 24).

APs and IPs endorsed CME credit and fulfillment of MOC requirements as the highest rated extrinsic benefits. There was no significant difference between the groups in these areas. These findings are not surprising considering the relative high time and cost associated with these efforts. Previous studies have shown similar results with regard to CME credit (11, 24, 28).

The only area that was rated significantly higher among IPs was financial compensation. Direct monetary payment to preceptors has shown mixed results in the literature. Multiple studies have demonstrated that financial compensation ranks among the lowest of potential reward methods (4, 16, 21). However, there was a selection bias in the recruitment methods of these studies: surveyed participants were APs who were typically *not* receiving financial compensation suggesting an implicit bias toward those who would devalue financial compensation. Furthermore, in a study by Peters and colleagues, retention of APs improved with increased financial compensation (22). The perceptions of participants in that study were revealing. Even though retention rates clearly improved with increasing reimbursement, participants stated that it was again the least significant factor in their decision to continue precepting students. The results suggest that compensation may be valued though underreported in the literature.

In our study, the role of financial compensation is further detailed when comparing whether APs or IPs were more or less likely to precept M1/M2 students and/or pediatrics residents. As shown in Table 3, APs were significantly more likely than IPs to work with an M1 or M2 student(s) *in addition to* precepting an M3 student in the pediatrics clerkship. This finding was curious due to disparities in reimbursement practices; namely, that preceptors are financially compensated when they work with M1 and M2 students but are not given any compensation when working with M3 students in pediatrics. Interestingly, more than a third of IPs who, by definition, were unwilling to precept an M3 student, were willing to precept an M1/M2 student suggesting financial motivation.

In summary, the role of financial compensation is complex and multifactorial based on the literature to date. The results of this study demonstrate that while it appears as though the majority of faculty members are not *exclusively* motivated by financial compensation, there is clearly a proportion that significantly values it; thus, financial incentives may increase IPs’ motivation to serve as a pediatric preceptor.

## Barriers

Time was the most significant barrier among IPs, with 71% noting it as a ‘significant’ or ‘very significant’ reason preventing their participation. However, previous studies have shown this is a common barrier even among APs (15, 18, 22), implying that time is an issue for all practicing physicians. It is not clear from this study whether APs have better resources or are perhaps simply more willing to sacrifice time to provide a preceptor experience.

## Implications for recruitment and retention

Intrinsic motivation is the major factor for APs to volunteer their time and for IPs to consider volunteering. Since these benefits are typically self-actualized, recruitment should begin simply by increasing awareness of the opportunities available to community preceptors. This may occur by distribution of brochures or mailings (15, 26), and emphasizing the implicit benefits awarded by precepting students.

Additionally, strong consideration should be given toward the development and provision of CME and MOC opportunities. Many institutions, including ours, offer community faculty the opportunity to claim AMA (American Medical Association) PRA Category 1 Credit by participating in CME offerings. In addition, the AMA permits all physicians to claim Category 2 Credit by supervising medical students and residents. However, the later opportunity is unhelpful from a recruitment standpoint, since the AMA prohibits organizations from advertising this fact or providing documentation of participation (29). The concept of offering MOC opportunities has not been explored in other studies, which likely reflects the relative infancy of this requirement. Based on the results of this survey, it may be worthwhile to explore the potential of creating institution-specific opportunities to satisfy these requirements.

In other studies, previous experiences; particularly with ‘good’ (22), ‘well prepared’ (11) students were the best predictors of a faculty member’s future desire to precept. Therefore, significant efforts should be made in the pre-clinical years and clinical orientations to prepare students as best as possible for the community pediatrics experience (11), ensuring a positive experience for the preceptor and student alike.

Our findings demonstrate that APs rated forms of appreciation, including letters, plaques, and funded social gatherings higher. This confirms the notion that APs often feel undervalued and underappreciated and emphasizes the importance of frequent praise and acknowledgment of support for the preceptorship time and commitment. Additionally, APs showed higher endorsement of feedback and educational faculty development opportunities than their inactive peers which confirms the value APs place on improving teaching skills. These findings are consistent with those of Ulltian and colleagues’ study which demonstrated a 90% retention rate among community faculty members surveyed at 10 medical schools (16). In their study, appreciation and faculty development were among the key drivers in promoting retention. In our study, neither appreciation nor faculty development opportunities were endorsed as highly by IPs. This suggests it may be beneficial to develop these programs not for recruitment, but for retention, of active community faculty preceptors.

## Limitations

We had a 28% response rate which introduces the possibility of non-response bias. However, surveys of physicians have significantly lower response rates than non-physicians and it is unclear whether response rate alone is a fair predictor of non-response bias (30).

In addition, our study was limited to pediatric physicians in the Richmond, Virginia metropolitan area. External validity is weak since we focused on participants within our local community.

Additional research is needed to determine how to overcome the barriers pediatric physicians face when precepting M3 students. Furthermore, a larger survey-based study should be administered nationwide to determine if the barriers and hurdles found in this study are consistent across the country. Finally, a prospective study could evaluate whether the recruitment and retention practices suggested in this study prove to be effective.

## Conclusions

Community pediatricians consider the intrinsic rewards of teaching medical students more valuable than extrinsic benefits. Efforts to recruit community preceptors should focus on encouraging awareness of the opportunities to teach and offering CME/MOC opportunities, if possible. Once recruited, retention may be improved by recognizing a preceptor’s value and providing opportunities for further faculty development. Pediatrics clerkship directors and administrators must recognize that time is the greatest barrier and should consider financial compensation to increase the pool of willing preceptors.
